# Effects of N Acetylcysteine on the Expression of Genes Associated with Reproductive Performance in the Goat Uterus during Early Gestation

**DOI:** 10.3390/ani12182431

**Published:** 2022-09-15

**Authors:** Kaibin Fu, Xiang Chen, Wei Guo, Zhinan Zhou, Yan Zhang, Taotao Ji, Peifang Yang, Xingzhou Tian, Weiwei Wang, Yue Zou

**Affiliations:** 1 Key Laboratory of Animal Genetics, Breeding and Reproduction in The Plateau Mountainous Region, Ministry of Education, Guizhou University, Guiyang 550025, China; 2 Key Laboratory of Animal Genetics, Breeding and Reproduction, Guiyang 550025, China; 3 College of Animal Science, Guizhou University, Guiyang 550025, China

**Keywords:** N acetylcysteine, RNA-seq, Qianbei Ma goat, uterus

## Abstract

**Simple Summary:**

The uterus is an important place for mammals to nurture new life, and improving the physiological function of the uterus is important for improving the reproductive efficiency of mammals. NAC is a small-molecule antioxidant with a positive regulatory effect on mammalian reproductive performance. We found that NAC can alter the expression of uterine genes in goats in early gestation. These DEGs may regulate uterine performance in early pregnancy in goats by participating in signalling pathways related to reproductive regulation, resistance to oxidative stress, immune regulation, angiogenesis and development, cytokines, and cell adhesion. These findings provide a fundamental reference for the modulation of reproductive performance in goats in early gestation by NAC.

**Abstract:**

N acetylcysteine (NAC) affects antioxidation and reactive oxygen species scavenging in the body and thereby promotes embryonic development and implantation and inhibits inflammation. The mechanism through which NAC regulates reproductive performance in the uteri of goats during early gestation remains unclear. In this study, the treatment group was fed 0.07% NAC for the first 35 days of gestation, whereas the control group received no NAC supplementation. The regulatory genes and key pathways associated with goat reproductive performance under NAC supplementation were identified by RNA-seq. RT–qPCR was used to verify the sequencing results and subsequently construct tissue expression profiles of the relevant genes. RNA-seq identified 19,796 genes coexpressed in the control and treatment groups and 1318 differentially expressed genes (DEGs), including 787 and 531 DEGs enriched in the treatment and control groups, respectively. A GO analysis revealed that the identified genes mapped to pathways such as cell activation, cytokine production, cell mitotic processes, and angiogenesis, and a KEGG enrichment analysis showed that the DEGs were enriched in pathways associated with reproductive regulation, immune regulation, resistance to oxidative stress, and cell adhesion. The RT–qPCR analysis showed that *BDNF* and *CSF-1* were most highly expressed in the uterus, that *WIF1* and *ESR2* showed low expression in the uterus, and that *CTSS*, *PTX3,* and *TGFβ-3* were most highly expressed in the oviduct, which indicated that these genes may be directly or indirectly involved in the modulation of reproduction in early-gestation goats. These findings provide fundamental data for the NAC-mediated modulation of the reproductive performance of goats during early gestation.

## 1. Introduction

Reproductive traits are among the most important economic traits of goats, and improving fertility is an important route for achieving high efficiency in the goat industry. A previous study showed that embryo loss can lead to a reduced litter size, longer litter intervals, and increased culling of breeding stock, resulting in great economic losses [1]. Improving the receptive and physiological environment of the uterus is an effective measure to prevent early embryo loss. The uterus is an important place for mammal reproduction, successful implantation of the embryo into the endometrium and maintenance of intrauterine environmental homeostasis during pregnancy are key steps in the successful Mammals reproduce [2,3]. Maintaining immune homeostasis between the embryo and mother during embryo attachment and pregnancy [4], enhancing antioxidant activity at the mother-foetus interface [5], and promoting placental angiogenesis and development [6] are essential. Of course, an adequate supply of nutrients is essential to ensure normal embryonic growth and development [7], and the digestion, absorption, and metabolism of nutrients such as protein, glucose, amino acids, and vitamins are necessary to support early embryonic growth and development [8,9]. It is thus clear that the conception of new life involves complex physiological regulation in the uterus. A previous study found that antioxidants improve endometrial receptivity, reduce reactive oxygen species (ROS) levels, and suppress the inflammatory response of the uterus [10]. Therefore, the effect of antioxidants on the mechanisms regulating reproductive performance in the goat uterus deserves further study.

N-acetylcysteine (NAC), a natural derivative of L-cysteine and precursor of reduced glutathione (GSH), is a common small-molecule antioxidant that promotes intracellular GSH biosynthesis and enhances glutathione-S-transferase activity and antioxidant effects [11]. The addition of trace amounts of NAC during oocyte maturation increases blastocyst rates, promotes early embryo formation, improves the success of early embryo development, and reduces embryo loss [12]. NAC improves the reproductive performance of animals [13], increases sperm counts and viability [14], and significantly regulates fertility in female rats, which results in the effective amelioration of gonadal hormone disorders and improvements in reproductive function by inhibiting the activation of NADPH oxidase [15]. In addition, NAC can prevent MXC-induced granulosa cell apoptosis and follicular atresia by reducing the oxidative stress induced by methoxychlor (MXC) and decreasing the ROS signalling pathway of apoptosis [16]. NAC can promote granulosa cell proliferation by reducing granulosa cell oxidative stress and apoptosis levels, and thereby restore ionizing radiation-induced ovarian and uterine functional damage and promote follicle development and embryo implantation [17]. Studies on mares have shown that the administration of NAC can support the antibiotic treatment of endometritis by reducing inflammation [18]. Additionally, NAC exerts a positive effect on the treatment of polycystic ovary syndrome (PCOS) [19]; specifically, NAC improves the receptivity and decidualization of the rat uterus and the level of gene expression during placental differentiation and is beneficial for foetal survival [20]. In addition, NAC improves production performance, reproductive performance, antioxidant status, immunity, and the delivery of maternal antibodies in Japanese quail under heat stress conditions [21]. In a previous study with Nubian goats, we found that different concentrations of NAC increase the lambing numbers of Nubian goats and that 0.07% NAC has the most significant effect [22]. In summary, we found that NAC plays an important role in the modulation of animal reproductive performance, but the molecular mechanisms through which NAC regulates reproductive performance in the goat uterus during early gestation are unclear.

Previous studies have shown that *CSF-1* can enhance endometrial regeneration by activating the PI3K/Akt signalling pathway [23], and that changes in the level of *CSF-1* expression in the uterus affect endometrial receptivity and the establishment of pregnancy [24]. *BDNF* and its receptors are key regulatory proteins in gonadal development, the physiological regulation of the ovary and uterus, and embryonic and placental development [25]. The *ESR2* gene is indispensable for female reproduction [26] and can regulate the physiological functions of the uterus by binding to oestrogen [27]. *TGF-β3* may control apoptosis and survival at specific stages of pregnancy [28], and maternal *TGF-β3* expression is downregulated during pregnancy, which may lead to miscarriage [29]. *PTX3* is essential for the maintenance of female fertility [30]. Changes in the *CTSS* expression levels are important for regulating endometrial and placental remodelling [31]. *WIF1* is involved in the regulation of reproductive performance by the Wnt signalling pathway, and *WIF1* overexpression may be related to the pathogenesis of preeclampsia [32].

The Qianbei Ma goat is an ideal goat breed in China with a high adaptability, good meat production performance and meat flavour, a high protein content, and other advantages, and this breed has considerable economic value and development potential [33]. A previous study showed that antioxidants can improve the meat quality and enhance antioxidant activity in the muscle of Qianbei Ma goats [34], but whether antioxidants can improve the reproductive performance of these goats remains to be determined. Therefore, building on the work of the previous study, 60 Qianbei Ma goats were employed in this study, separated into two groups (control and NAC groups), and then fed for 35 days. RNA-seq and bioinformatics analyses were performed to screen key candidate genes and signalling pathways affecting the reproductive performance of goats during early gestation, and to investigate the changes in the genome-wide gene expression levels in early-gestation goats. In conjunction with previous studies, we constructed tissue expression profiles of the *TGFβ-3*, *CSF-1*, *BDNF*, *PTX3*, *CTSS*, *WIF1,* and *ESR2* genes by RT–qPCR with the aim of providing a reference for elucidating the molecular mechanisms through which NAC regulates reproductive performance in early-gestation goats.

## 2. Materials and Methods

### 2.1. Experimental Animals

All Qianbei Ma goats used in the trial originated from the same farms (Fuxing Herding Co., Zunyi, China) (106.198244 E, 28.26403 N). The farm’s Qianbei Ma goats are sexually mature at 4 months of age and ready for mating at approximately 8 months of age. Does enter oestrus throughout the year and have an oestrus cycle of 19–21 d, an oestrus duration of 24–48 h, and a litter rate of approximately 200%. Sixty healthy unpregnant goats (aged 2–3 years; 32.38 ± 3.12 kg, mean ± SEM) raised in the same environment were employed in this study. Prior to the experiments, these Qianbei Ma goats were housed at a Fuxing Herding Co., Ltd., sheepfold under constant temperature (25 °C) and a fixed light/dark cycle (12 h/12 h) to achieve as similar a physiological state as possible. After acclimatization, vaginal implants impregnated with progesterone were used for 12 d to synchronize the oestrus. After removal of the implants, equine chorionic gonadotropin (eCG, 330 IU/each) and prostaglandin (PG, 1 mL/each) (Sansheng Biologicals, Ningbo, China) were injected intramuscularly. After 48 h of concentrated oestrus, the does were inseminated for the first time with fresh semen from six healthy bucks and for the second time 12 h later. Both inseminations were performed using the vaginal opener method with a volume of 0.5 mL per dose. The ejaculate volume per goat was 0.8–1.5 mL, the sperm viability was about 70%, and the semen was diluted 1:4 with saline for insemination. According to a completely randomized design, 60 Qianbei Ma goats were divided into a control group (basal diet, n = 30) and a treatment group (basal diet + 0.07% NAC, n = 30) (Fanhai Biotechnology Co., Ltd., Zhuhai, China). Each goat was fed regularly and quantitatively in the sheepfold (feeding time: 9:00, 17:00) and had free access to water. After 35 days of feeding under the same environmental conditions and nutrition levels, pregnancy was determined using a Micro-imager 1000 sector ultrasound scanner equipped with a 3.5-MHz abdominal transducer (Ausonics, Sydney, Australia), and three pregnant goats in the treatment and control groups were randomly selected for euthanasia according to their ear tag numbers. The number of embryos in the euthanized goats was recorded. To ensure consistency with previous work, the experimental goats were fed a previously described basal diet [22]. Specific information is provided in Table 1.

### 2.2. Sample Collection

Does in the treatment and control groups were euthanized at day 35 of gestation. Gonadal axis tissue samples were collected from the goats within 20 min after euthanasia and washed with phosphate-buffered saline solution. All the samples were then snap frozen in liquid nitrogen and subsequently transferred to storage at −80 °C for further analysis.

### 2.3. Total RNA Extraction and RNA Sequencing

Total RNA was extracted from the gonadal axis tissues of the control and treated groups using TRIzol reagent (Invitrogen, Carlsbad, CA, USA) and an RNeasy RNA purification kit containing DNase treatment (Qiagen, Valencia, CA, USA) according to the manufacturer’s instructions. The RNA concentration and purity were determined using a 2100 Bioanalyzer (Agilent Technologies, Santa Clara, CA, USA), and the RNA quality was assessed by 1% agarose gel electrophoresis. High quality RNA with an OD260/280 absorbance ratio in the range of 1.8–2.0, an RNA integrity > 7.0, and a 28S:18S ratio > 1.0 was used for sequencing on the Illumina NovaSeq 6000 system to generate 150-bp paired-end reads.

Image data of sequenced fragments obtained from high-throughput sequencers were converted into sequence data (reads) by CASAVA base identification to generate files in fastq format. The RNA-seq fastq raw data were then filtered using Fastp v to remove reads with adapters, N-containing reads, and low-quality reads (quality scores < 20) and thus obtain clean data. HISAT2 (v2.0.5) was used to map the clean reads to the goat (Capra hircus) (ARS1.2) reference genome [35]. FeatureCounts (1.5.0-p3) was then used to calculate the number of reads mapped to each gene to estimate the expression of each gene transcript. Gene expression levels were estimated based on fragments per kilobase million mapped reads (FPKM) values [36]. In this experiment, we used DESeq2 software (1.20.0) based on the negative binomial distribution model to perform differential expression analysis between the treatment and control groups. The method described by Benjamini and Hochberg was used to adjust the resulting *p* value (P-adj) and thus control the false discovery rate. Genes with a |log2-fold change| ≥ 1 and P-adj < 0.05 were selected as significantly differentially expressed genes (DEGs) [37]. Analyses of the enrichment of all identified DEGs in GO functions and KEGG pathways were performed using clusterProfiler (3.8.1) software, and the results were considered significant if *p* < 0.05.

### 2.4. Quantitative Reverse Transcription PCR

DEGs associated with reproduction and immunity were selected from the sequencing results, and their expression levels were verified by quantitative reverse transcription PCR (RT–qPCR). One thousand nanograms of total RNA was synthesized into complementary DNA (cDNA) using the RT Master Mix for qPCR II kit (MCE, Monmouth Junction, NJ, USA). The synthesis reaction conditions were 25 °C for 5 min, 55 °C for 15 min, and 85 °C for 2 min. Information on the primers used for RT–qPCR is shown in Table 2. Relative gene expression levels were measured using the CFX96 Real-Time PCR system (BioRad, Foster City, CA, USA). The reaction system consisted of a volume of 10 μL and included the following components: 5 μL of 2 × RealStar Green Fast Mixture, 0.5 μL of each forward and reverse primer (10 pmol/μL), 1 μL of cDNA (1000 ng/μL), and 3 μL of ddH_2_O. The reaction conditions were as follows: 1 cycle at 95 °C for 2 min, followed by 40 cycles at 95 °C for 15 s and annealing temperature (see Table 2 for details) for 30 s, and 72 °C for 30 s. The melting curve was set automatically by the machine (base temperature 65 °C, rising by 0.5 °C every 5 s to 95 °C). The annealing temperature of β-actin is the same as the annealing temperature of each gene. The specificity of the PCR primers was confirmed through a single peak in the melting curve. The amplification efficiency of each pair of primers was in the range of 90–110% at different cDNA template concentrations. Three replicates were performed, and the average value was reported as the result. The gene expression levels were normalized to β-actin expression, and relative gene expression levels were calculated via the 2^−ΔΔCt^ method [38].

### 2.5. Statistical Analysis

Differences in the relative expression levels of DEGs were analysed using SPSS 18.0 software (IBM Corporation, Armonk, NY, USA), and differences in reproductive performance were analysed using a Student’s t test and considered significant if *p* < 0.05. The relative expression levels of the genes are presented as histograms. The results for reproductive performance are expressed as the mean ± standard deviation (SD).

## 3. Results

### 3.1. Effect of N-Acetylcysteine on the Reproductive Performance of Qianbei Ma Goats

To verify the effect of adding NAC to the diet on the reproductive performance of Qianbei Ma goats, the litter size and conception rate of the control and treatment groups were compared (Table 3). The treatment group consisted of 23 pregnant does, the total litter size was 54 (the number of goat embryos sampled for slaughter was 7), and the average litter size was 2.35. The control group comprised 21 pregnant does, the total litter size was 45 (the number of goat embryos sampled for slaughter was 5), and the average litter size was 2.14. No stillbirths were recorded in either the treatment or control groups, and all the foetuses were eutocic. The number of litters in the treatment group was increased by 0.21 compared with that in the control group, but the difference was not significant (*p* > 0.05). The conception rate of the treatment group was increased by 6.67% compared with that of the control group.

### 3.2. Sequence Quality and Differential Gene Expression Profiling

**Figure 1:**
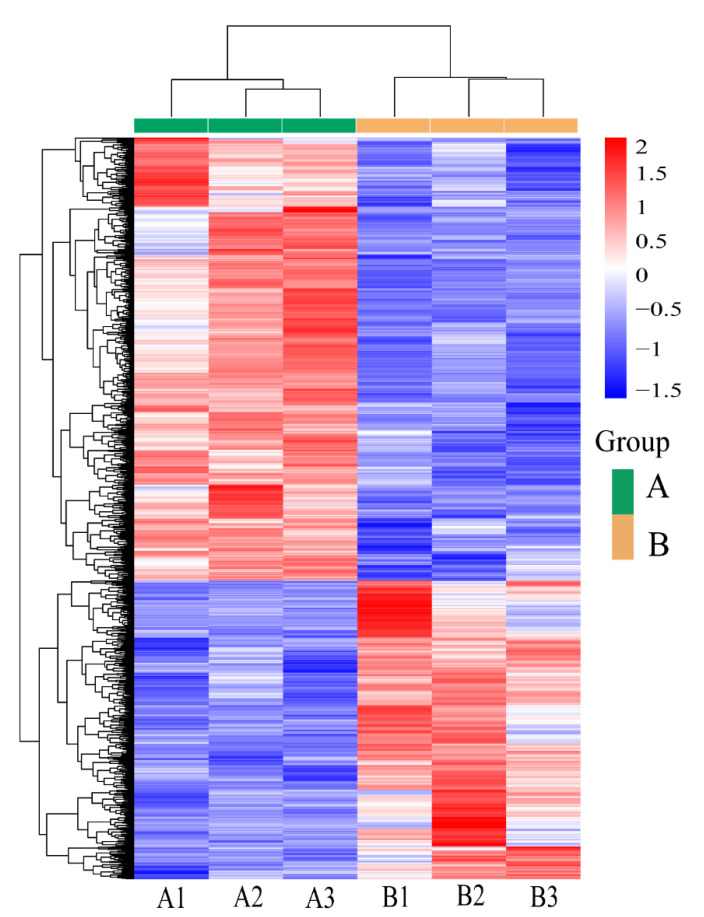
Clustering heat map of DEGs among the samples. The abscissa is the sample name, and the ordinate is the normalized value of the differential gene FPKM (Z-score). The redder the colour, the higher the expression level, and the bluer the colour, the lower the expression level.

To obtain comprehensive gene expression profiles of the uterine horns collected from the control and treatment groups, six RNA-seq libraries were constructed. RNA-seq analysis revealed averages of 4,682,911 and 49,335,290 raw reads in the control and treatment groups, respectively, and the sequencing error rates ranged from 0.02% to 0.03%. After quality control screening, the final average numbers of clean reads obtained for the control and treatment groups were 44,978,308 and 47,403,637, respectively. In addition, the GC content of each sample averaged 51.89%, all Q20 values were greater than 97.58%, and all Q30 values were higher than 93.5%. The percentage of clean reads in each sample mapped to the goat reference genome was above 96.07%, indicating a high level of sequencing accuracy. After quality control and mapping to a reference genome, gene expression levels were obtained for the different samples. The gene expression profiles showed similar expression patterns within each group of samples and differed between groups of samples, suggesting overall transcriptional differences between the control and treatment groups (Figure 1) and indicating that the obtained biological information could be used for subsequent analysis.

### 3.3. Screening of DEGs

**Figure 2:**
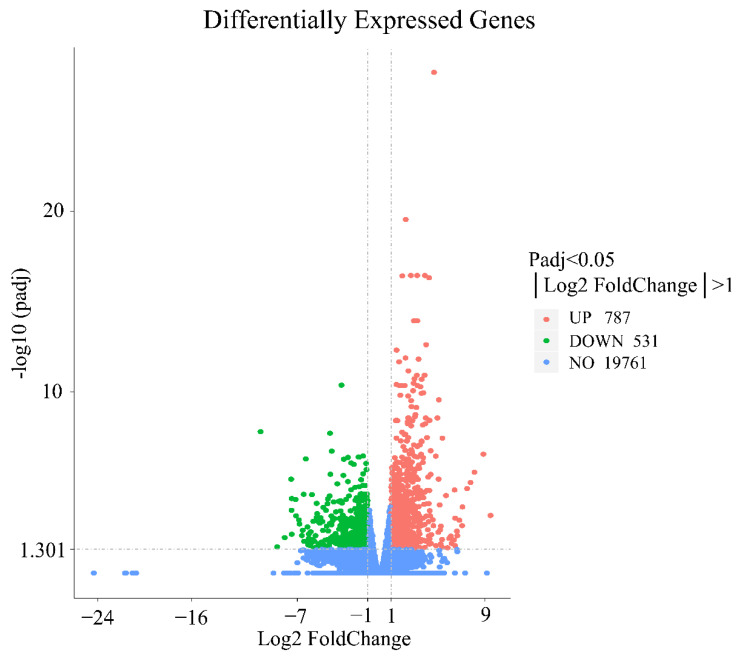
Differential mRNA expressions between the treatment and control groups. Red dots indicate significantly upregulated genes, green dots indicate significantly downregulated genes, and blue dots indicate genes with no difference.

A comparative analysis revealed that 19,761 genes were coexpressed in the control and treatment groups. Using a |log2-fold change| ≥ 1 and P-adj < 0.05 as the screening criteria for DEGs, a total of 1318 DEGs were identified in the control and experimental groups, and these included 787 genes in the experimental group and 531 genes in the control group (Figure 2).

### 3.4. GO Function and KEGG Enrichment Analyses

**Figure 3:**
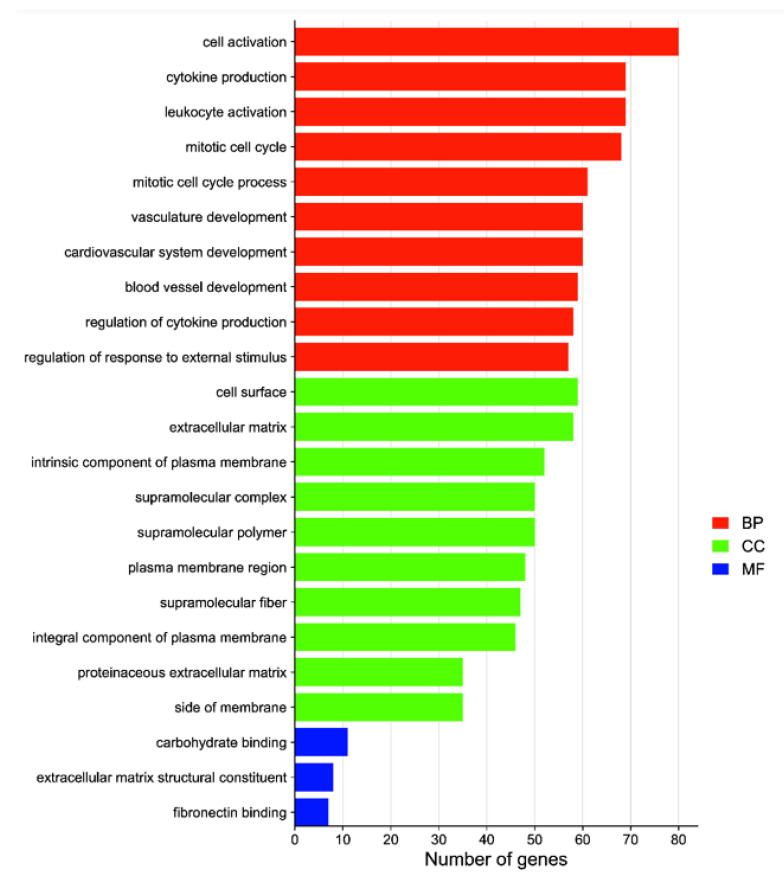
GO enrichment analysis of DEGs between the treatment and control groups. The vertical coordinate indicates the enriched GO term, and the horizontal coordinate indicates the number of DEGs in that term. Abbreviations: biological processes (BP), cellular components (CC), molecular functions (MF).

**Figure 4:**
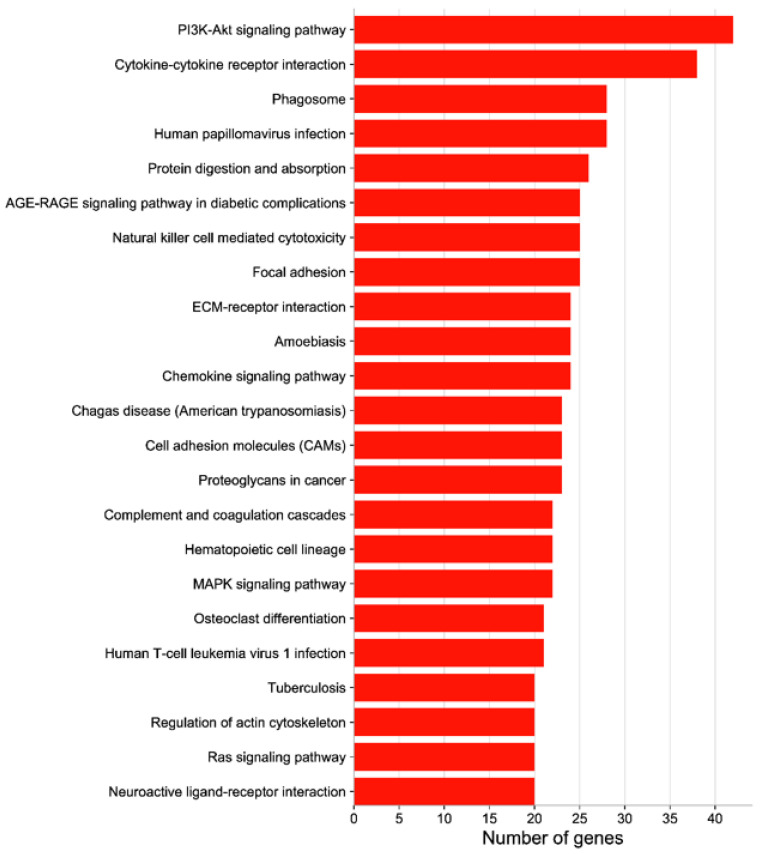
KEGG enrichment analysis of DEGs between the treatment and control groups. The vertical coordinate indicates the KEGG pathway, and the horizontal coordinate indicates the number of DEGs enriched to this pathway.

**Figure 5:**
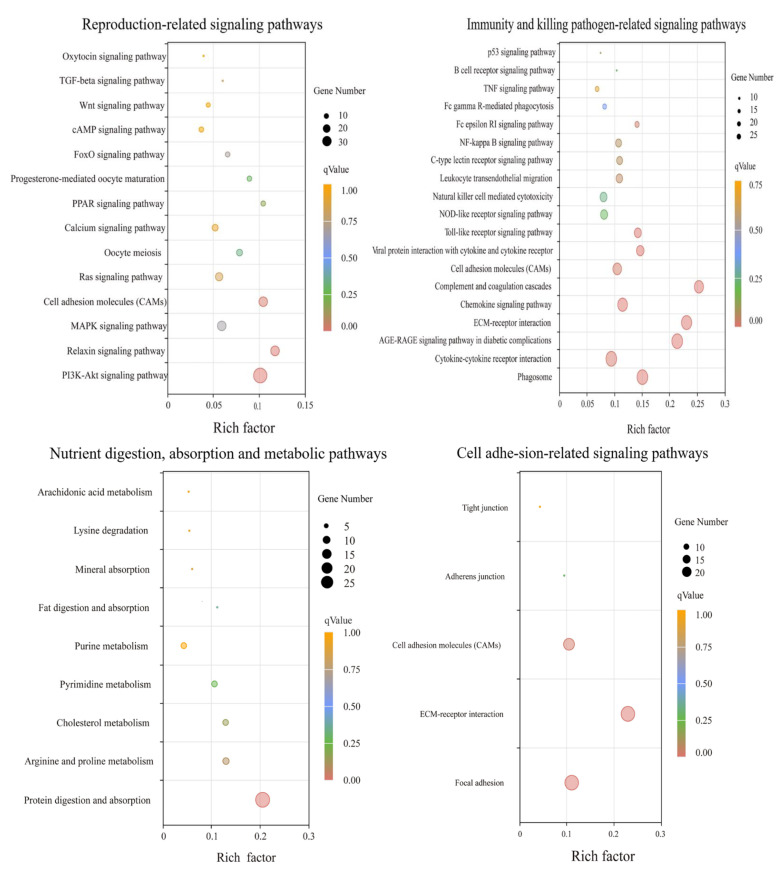

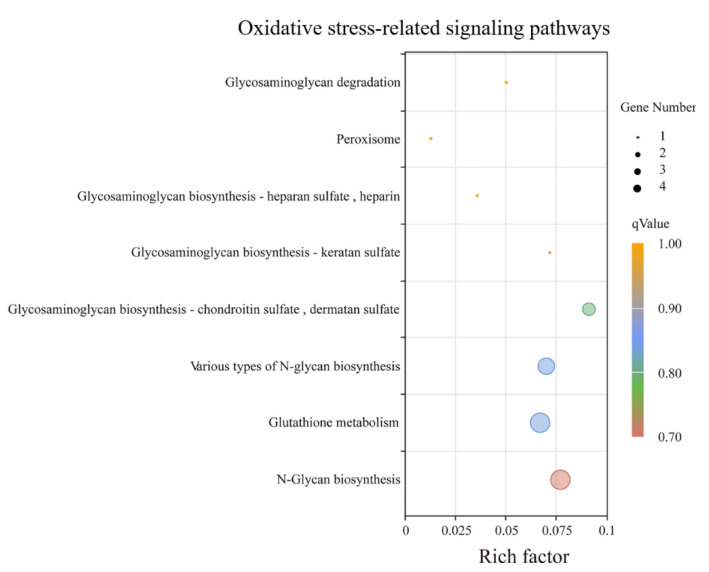
The results of KEGG enrichment analyses based on the upregulated genes in the treatment group compared to the control group.

GO functional clustering analysis was performed with the 1318 screened DEGs, which were divided into 3 major categories and 295 subcategories: biological processes accounted for 82.37% of the enriched DEGs, cellular components accounted for 16.61%, and molecular functions accounted for 1.02%. The GO analysis showed the enrichment of more DEGs in the pathways of cell activation, cytokine production, leukocyte activation, mitotic cycle, mitotic cycle process, development of the vascular system, and development of the cardiovascular system (Figure 3). The 1318 DEGs were also annotated in the KEGG database: 272 signalling pathways were enriched in the treatment group, with 32 significant differences, whereas 238 signalling pathways were enriched in the control group, with 4 significant differences. The greatest numbers of DEGs were enriched in the PI3K/Akt signalling pathway, cytokine–cytokine receptor interaction, phagosome, protein digestion and absorption, and other KEGG signalling pathways (Figure 4). The analysis revealed that several signalling pathways related to reproduction, immunity and inactivation of pathogenic bacteria, nutrient digestion and uptake, resistance to oxidative stress, and cell adhesion were enriched in the test group (Figure 5).

### 3.5. Analysis of DEGs Patterns

**Figure 6:**
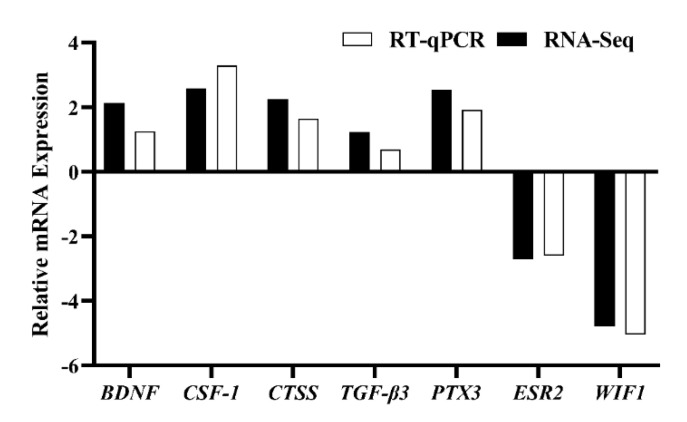
Validation of DEGs by RT–qPCR.

**Figure 7:**
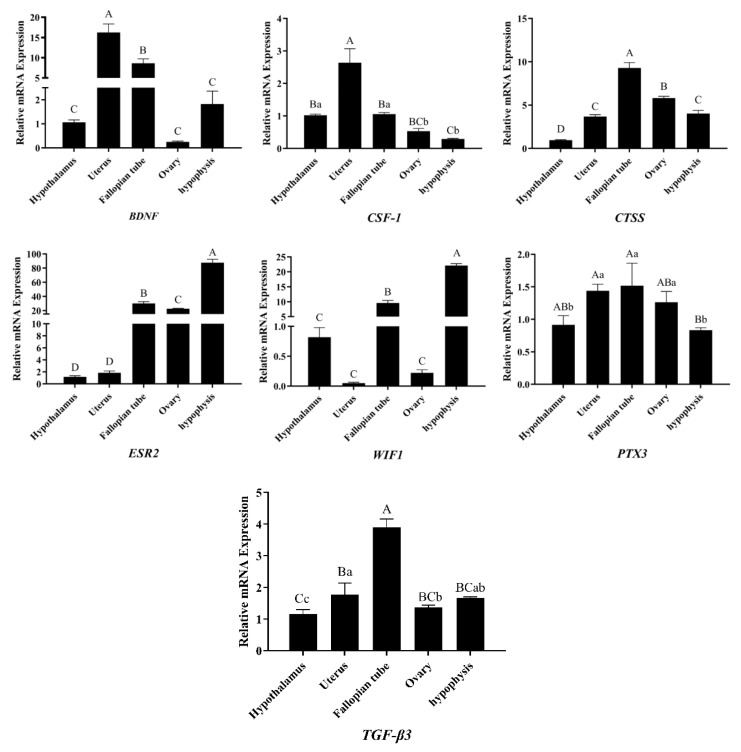
Differential gene expression patterns in the gonadal axis tissue of the Qianbei Ma goats. Different capital letters A, B, C and D indicate highly significant differences in expression levels between different tissues (*p* < 0.01) and different lower case letters a, b and c indicate significant differences in expression levels between different tissues (*p* < 0.05).

To check the accuracy of the RNA-seq results, we verified the changes in the mRNA expression levels of upregulated genes (*CTSS*, *TGFβ-3*, *CSF-1*, *BDNF*, and *PTX3*) and downregulated genes (*ESR2* and *WIF1*), and the results were consistent with the RNA-seq results (Figure 6), indicating that the sequencing results of RNA-seq were reliable. After NAC treatment, we constructed tissue expression profiles of these genes in the gonadal axis of Qianbei Ma goats in early pregnancy. The results showed that after NAC supplementation, these genes were expressed in the gonadal axis of early-gestation Qianbei Ma goats (Figure 7); the relative expression levels of the *BDNF* and *CSF-1* genes were highest in the uteri of Qianbei Ma goats, and the differences from the other gonadal axis tissues were highly significant (*p* < 0.01). *WIF1* showed the lowest relative expression level in the uterus, followed by the ovary, and *ESR2* presented the lowest relative expression level in the hypothalamus, followed by the uterus. *TGF-β3*, *CTSS,* and *PTX3* presented the highest expression levels in the fallopian tube; the patterns of expression in descending order were as follows: *TGF-β3*, fallopian tube > uterus > pituitary gland > hypothalamus > ovary; *CTSS*, fallopian tube > ovary > uterus > pituitary > hypothalamus; and *PTX3*, fallopian tube > uterus > ovary > hypothalamus > pituitary.

## 4. Discussion

Embryo attachment is a complex biological process that is simultaneously regulated by several factors, among which immune-related processes between the maternal uterus and the embryo before and during implantation are an essential component of pregnancy establishment [39]. In mammals, the successful implantation of the embryo into the maternal endometrium is a critical step in pregnancy [40]. NAC can suppress the inflammatory response of the body by scavenging reactive oxygen radicals and inhibiting the increases in NF-κB activity induced by TNF-α, IL-1β, and LPS in vivo, and thereby enhance the antioxidant capacity of the body [41]. In this study, 0.07% NAC was fed to Qianbei Ma goats in early pregnancy to investigate the effect of NAC on their reproductive performance. Our preliminary data suggest that NAC can increase the conception rate of Qianbei Ma goats. In a previous study with Nubian goats, we found that NAC significantly increases the litter size [22]. However, our available data suggest that NAC can increase the litter size of Qianbei Ma goats, but the increase is not significant. This result may be largely related to the reproductive rate of the goats, because the reproductive rate of our subjects was higher than that of the Nubian goat subjects, and it is speculated that NAC may exert a greater effect on goat populations with a low reproductive rate than on goat populations with a high reproductive rate. However, given the limitations of our sample size, our findings can only be used as a general reference. There is a need to expand the sample size of the experimental population in the next study to further elucidate the specific regulatory mechanisms underlying the effects of NAC on goat reproductive performance. Overall, these data suggest that the addition of 0.07% NAC to goat rations in early gestation may have some effect on improving the reproductive rates of goats and can be used as a general reference. By RNA-seq, we identified a total of 1318 DEGs in the control and treatment groups, including 787 DEGs in the treatment group and 531 DEGs in the control group. This finding suggests that NAC feeding may be an important factor affecting gene expression in early-gestation Qianbei Ma goats.

The completion of important physiological processes in living organisms often requires the co-regulation of multiple genes. Therefore, enrichment analyses of gene functions to identify biological pathways that play a key role in biological processes are important to reveal and understand the underlying molecular mechanisms of biological processes. We performed a KEGG enrichment analysis of DEGs in the treatment and control samples. Several reproduction-related signalling pathways were enriched in the treatment group, which suggested that NAC may play an important regulatory role in the reproductive performance of goats. Multiple signalling pathways associated with immunity and the inactivation of pathogenic bacteria were enriched in the treatment group, implying that the addition of NAC to the diet may improve the ability of goats to resist pathogenic bacteria in early pregnancy, regulate the immune balance between the maternal uterus and the foetus, and thus maintain normal foetal growth and development. Previous studies also suggest that maintaining maternal and foetal immune regulation during pregnancy [42] and improving maternal resistance to pathogenic bacteria during pregnancy are important for normal embryonic development [43]. In addition, several signalling pathways related to the digestion, absorption, and metabolism of proteins, amino acids, minerals, and other nutrients were enriched in the treatment group; these results suggest that NAC could improve the digestion, absorption, and utilization of nutrients in does during early pregnancy and thus ensure that the nutritional needs for normal foetal growth and development are met during pregnancy. Importantly, however, peroxisomes, glutathione metabolism, and N-glycan biosynthesis, which are pathways associated with resistance to oxidative stress, were enriched in the treatment group; these results indicate that NAC feeding results in a greater antioxidant capacity of goats in early gestation. Studies have found that increasing the maternal antioxidant capacity during pregnancy is effective for increasing the litter size in sows [44] and reducing the levels of maternal, placental, and foetal inflammation [45]. In addition, several pathways related to cell adhesion were enriched in the treatment group, suggesting that NAC could improve embryo attachment in the maternal uterus and reduce pregnancy failure due to insufficient embryo attachment. Cytokines facilitate the exchange of information between cells [46], are important regulators of pregnancy and delivery, and play an important role in promoting healthy maternal pregnancy and foetal development [47]. A KEGG analysis identified cytokine–cytokine receptor interactions enriched in many DEGs; interestingly, the GO analysis also identified many DEGs enriched in cytokine production. This finding indicates that in the presence of NAC, cytokines may be an important factor in the reproductive performance of goats. Previous studies have shown that mitosis and angiogenesis are beneficial for promoting embryo attachment and development [48,49]. Moreover, the GO analysis revealed that many DEGs were enriched in cellular mitotic processes and in pathways of angiogenesis and development, which suggested that NAC may be beneficial for improving pregnancy in goats.

*FPR2* has been found to trigger trophoblast dysfunction through the PI3K/AKT signalling pathway, leading to recurrent spontaneous abortions [50]. The PI3K/Akt signalling pathway affects embryo implantation by regulating the expression of *RhoA* [51]. *NM23* can affect metaphase by regulating mouse and human endometrial stromal cells via the PI3K-Akt-mTOR signalling pathway [52]. miR-494-3p regulates endometrial receptivity in mice via the PI3K/AKT/mTOR pathway [53]. Our preliminary data suggest that the PI3K/Akt signalling pathway is enriched in the most DEGs compared with other signalling pathways. Therefore, we analysed the DEGs enriched in the PI3K/Akt signalling pathway and found that most of them are involved in regulation related to mammalian reproductive performance. For example, the knockdown of *FN1* inhibits the PI3K/Akt signalling pathway, whereas its overexpression activates the PI3K/Akt signalling pathway to inhibit apoptosis in human trophoblast cells [54]. *CSF-1R* directly regulates the preimplantation development and innate immune function of trophoblast cells, and *CSF-1R* deficiency in mice results in embryonic death [55]. Using RNA-seq technology, Armstrong et al. revealed that *EREG* expression is significantly associated with placental morphology [56]. *IFNAR1* in the pig endometrium and foetus may establish and maintain pregnancy by mediating the action of type I interferon during implantation [57]. *IGF1* plays crucial roles in the regulation of sexual development and reproduction in mammals [58]. Numerous studies have shown that members of the integrin family are highly expressed in the gonadal axis of female animals and play an important role in embryonic attachment [59,60,61], and the present study showed that the *ITGAM*, *ITGAV*, *ITGA1*, *ITGA5*, *ITGB1,* and *ITGB2* integrin families are highly expressed in the treatment group, which suggests that integrins play an important role in the modulation of reproduction in does. *KRAS* plays an important role in regulating the implantation process in mouse embryos [62]. It has been reported that miR-183 can inhibit embryo implantation by binding to *LAMC1* [63]. The regulation of *VEGFC* gene expression in the bovine uterus during implantation affects the maternal uterine recognition of pregnancy and vascular remodelling prior to implantation [64]. The abovementioned previous studies have shown that the *FN1*, *CSF*-*1R*, *EREG*, *IFNAR1*, *IGF1*, *ITGAM*, *ITGAV*, *ITGA1*, *ITGA5*, *ITGB1*, *ITGB2*, *KRAS*, *LAMC1,* and *VEGFC* genes play important roles in mammalian reproduction. Notably, we found that the PI3K/Akt signalling pathway was similarly screened after the feeding of Nubian goats with NAC [22]. Therefore, we speculate that the PI3K/Akt signalling pathway may be an important pathway through which NAC regulates uterine performance in goats.

Based on the RNA-seq data and the results from previous studies, we screened the *TGFβ-3*, *CSF-1*, *BDNF*, *PTX3*, *CTSS*, *WIF1,* and *ESR2* genes, verified the expression of these genes in the gonadal axis of Qianbei Ma goats by RT–qPCR, and found that all of these genes were expressed in the gonadal axis of Qianbei Ma goats under the action of NAC. Studies have shown that *BDNF* can promote oocyte maturation and early embryonic development [65]. Uterine *CSF-1* mRNA expression and synthesis promote placental growth and differentiation [66] and help to establish and maintain a normal pregnancy [67]. In this study, *BDNF* and *CSF-1* were found to be enriched in the PI3K/Akt signalling pathway and were highly expressed in the uterus. This finding indicates that *BDNF* and *CSF-1* may play a role in the uteri of Qianbei Ma goats in early pregnancy through the PI3K/Akt signalling pathway, and thus regulate the reproductive performance of early-pregnancy Qianbei Ma goats. The Wnt signalling pathway plays an important role in mammalian reproduction [68]. *WIF1* acts as an antagonist of the Wnt signalling pathway and inhibits its activation [69]. This study showed that *WIF1* was expressed at low levels in the uterus of Qianbei Ma goats in early gestation, which suggests that the low expression of *WIF1* may activate the Wnt signalling pathway to maintain pregnancy. The expression of *ESR2* mRNA in the uterus during implantation and early gestation is extremely low [70] and decreases during pregnancy [71], which is consistent with the results of the present study showing that the *ESR2* gene is expressed at low levels in the uteri of Qianbei Ma goats in early gestation. The fallopian tubes are not only the place where the sperm and egg are united, but secretions from the fallopian tubes also play an active role in improving endometrial receptivity, facilitating embryo implantation and development, and maintaining pregnancy [72]. Our data show that *TGF-β3*, *CTSS,* and *PTX3* are most highly expressed in the oviducts of Qianbei Ma goats, which suggests that high expression of *TGF-β3*, *CTSS,* and *PTX3* in the oviducts may promote the secretion of certain substances that directly or indirectly affect embryogenesis and development.

## 5. Conclusions

Dietary supplementation with NAC alters the expression of uterine horn genes in goats in early gestation. These DEGs are involved in signalling pathways related to reproductive regulation, immune regulation, resistance to oxidative stress, angiogenesis and development, cytokines, and cell adhesion. Notably, activation of the PI3K/Akt signalling pathway and altered expression levels of *CSF-1*, *BDNF*, *WIF1*, *ESR2*, *TGF-β3*, *CTSS,* and *PTX3* genes may be important mechanisms through which NAC regulates uterine performance in goats in early gestation.

## Figures and Tables

**Table 1:** Ingredients and nutrient composition of experimental diets (DM basis).

Ingredients %	Content	Chemical Composition %	Content
Corn silage	50	DM	60.13
Chinese wildrye	20	Metabolic energy (MJ/kg DM)	12.51
Corn	15	Crude protein, DM	13.42
Soybean meal	8.02	Organic matter	86.38
Wheat bran	4.98	NDF	38.67
Calcium bicarbonate	0.5	ADF	31.07
Sodium chloride	0.5	Ca	0.68
Premix *	1	P	0.49
Total	100		

* Per kilogram of premix of the diet contains vitamin A 55,000 IU, vitamin D 11,500 IU, vitamin E 13,000 IU, MgSO_4_·H_2_O 110 g, CuSO_4_·5H_2_O 0.7 g, FeSO_4_·7H_2_O 3.0 g, MnSO_4_·H_2_O 2.5 g, ZnSO_4_·H_2_O 5.0 g, Na_2_SeO_3_ 15 mg, KI 40 mg, CoCl_2_·6H_2_O 28 mg. DM, dry matter; NDF, neutral detergent fibre; ADF, acid detergent fibre.

**Table 2:** Details of primers for RT-qPCR validation of mRNA sequencing data.

Gene Name	Primer Sequences (5′→3′)	GenBank ID	Product Size/bp	Tm/°C
*BDNF*	F: GTCCTTGAAAAAGTCCCCG R: CTATCCGAATGAACCGCCA	XM_005690025.2	205	61
*CSF-1*	F: AGGTGTCGGAGAACTGTAGC R: TTGGGGGTGTTGTCTTTGAA	XM_013962557.2	214	56
*CTSS*	F: ACTGGAGAGAGAAGGGGTGT R: ATGGCTTTGTAGGGATAGGA	XM_005677657.3	268	59.4
*PTX3*	F: CTGGTCGCTGATGCTGT R: GCCATTCTTTTCTTGCC	XM_018048475.1	183	59.4
*TGF-β3*	F: GCCAAGCAGCGGTAT R: GCAAGAGCCATTCACG	XM_005686141.3	100	57.9
*WIF1*	F: GTGGCAGCATTTGAAGTGAAC R: ATCCATCAGGACATTCGCAG	XM_005680217.2	172	56
*ESR2*	F: GACAGACCACAAGCCCAAA R: GGCACAACTGCTCCCACTA	NM_001285688.1	191	60
*β-actin*	F: TGATATTGCTGCGCTCGTGGT R: GTCAGGATGCCTCTCTTGCTC	XM_018039831.1	189	All

**Table 3:** The effect of NAC on the reproductive performance of Qianbei Ma goats.

Items	Treatment Group	Control Group
No. of test does	30	30
No. of pregnant goats	23	21
No. of total kids	54	45
No. of kids in slaughtered does	7	5
No. of average kids	2.35 ± 0.71	2.14 ± 0.73
Conception rate	76.67%	70%

## Data Availability

The raw data supporting the conclusions of this article will be made available by the authors, without undue reservation. Additionally, our RNA-seq sequencing data have been deposited in a public repository and are available from this website at: https://dataview.ncbi.nlm.nih.gov/object/PRJNA864595?reviewer=p3jrusgudh7epsjnfiqbmin3g7. (Date of visit: 1 January 2025).

## References

[1] Jonker F.H. (2004). Fetal death: Comparative aspects in large domestic animals. Anim. Reprod. Sci..

[2] Bazer F.W., Burghardt R.C., Johnson G.A., Spencer T.E., Wu G. (2018). Mechanisms for the establishment and maintenance of pregnancy: Synergies from scientific collaborations. Biol. Reprod..

[3] Bowen J.A., Burghardt R.C. (2000). Cellular mechanisms of implantation in domestic farm animals. Semin. Cell Dev. Biol..

[4] Johns D.N., Lucas C.G., Pfeiffer C.A., Chen P.R., Meyer A.E., Perry S.D., Spate L.D., Cecil R.F., Fudge M.A., Samuel M.S. (2021). Conceptus interferon gamma is essential for establishment of pregnancy in the pig. Biol. Reprod..

[5] Hussain T., Murtaza G., Metwally E., Kalhoro D.H., Kalhoro M.S., Rahu B.A., Sahito R., Yin Y., Yang H., Chughtai M.I. (2021). The role of oxidative stress and antioxidant balance in pregnancy. Mediators Inflamm..

[6] Maltepe E., Fisher S.J. (2015). Placenta: The forgotten organ. Annu. Rev. Cell Dev. Biol..

[7] Mullen M.P., Elia G., Hilliard M., Parr M.H., Diskin M.G., Evans A.C., Crowe M.A. (2012). Proteomic characterization of histotroph during the preimplantation phase of the estrous cycle in cattle. J. Proteome Res..

[8] Bazer F.W., Kim J., Ka H., Johnson G.A., Wu G., Song G. (2012). Select nutrients in the uterine lumen of sheep and pigs affect conceptus development. J. Reprod. Dev..

[9] Cetin I., Berti C., Calabrese S. (2010). Role of micronutrients in the periconceptional period. Hum. Reprod. Update.

[10] Zheng P., Qin X., Feng R., Li Q., Huang F., Li Y., Zhao Q., Huang H. (2022). Alleviative effect of melatonin on the decrease of uterine receptivity caused by blood ammonia through ROS/NF-κB pathway in dairy cow. Ecotoxicol. Environ. Saf..

[11] Whitaker B.D., Casey S.J., Taupier R. (2012). N-acetyl-l-cysteine supplementation improves boar spermatozoa characteristics and subsequent fertilization and embryonic development. Reprod. Domest. Anim..

[12] Whitaker B.D., Knight J.W. (2010). Effects of N-acetyl-cysteine and N-acetyl-cysteine-amide supplementation on in vitro matured porcine oocytes. Reprod. Domest. Anim..

[13] Sun W.S., Jang H., Park M.R., Oh K.B., Lee H., Hwang S., Xu L.J., Hwang I.S., Lee J.W. (2021). N-acetyl-L-cysteine improves the developmental competence of bovine oocytes and embryos cultured in vitro by attenuating oxidative damage and apoptosis. Antioxidants.

[14] Rahil J., Kazem P., Hayati R.N., Hossein N.M. (2019). Effects of N-acetyl-cysteine supplementation on sperm quality, chromatin integrity and level of oxidative stress in infertile men. Reprod. Biol. Endocrin..

[15] Helal M.A. (2016). The effects of N-acetyl-L-cysteine on the female reproductive performance and nephrotoxicity in rats. Ren. Fail..

[16] Bhardwaj J.K., Saraf P. (2017). N-acetyl cysteine-mediated effective attenuation of methoxychlor-induced granulosa cell apoptosis by counteracting reactive oxygen species generation in caprine ovary. Environ. Toxicol..

[17] Wei G., Jin-Xiao L., Chi M., Jing-Yin D., Qiu Y. (2017). The protective effect of N-Acetylcysteine on ionizing radiation induced ovarian failure and loss of ovarian reserve in female mouse. Biomed Res. Int..

[18] Witte T.S., Melkus E., Walter I., Senge B., Schwab S., Aurich C., Heuwieser W. (2012). Effects of oral treatment with N-acetylcysteine on the viscosity of intrauterine mucus and endometrial function in estrous mares. Theriogenology.

[19] Rafiee B., Karbalay-Doust S., Tabei S., Azarpira N., Alaee S., Lohrasbi P., Bahmanpour S. (2022). Effects of N-acetylcysteine and metformin treatment on the stereopathological characteristics of uterus and ovary. Eur. J. Transl. Myol..

[20] Hu M., Zhang Y., Ma S., Li J., Wang X., Liang M., Sferruzzi-Perri A.N., Wu X., Ma H., Brännström M. (2021). Suppression of uterine and placental ferroptosis by N-acetylcysteine in a rat model of polycystic ovary syndrome. Mol. Hum. Reprod..

[21] Omid K., Amirali S., Ahmad K. (2018). N-Acetyl cysteine improves performance, reproduction, antioxidant status, immunity and maternal antibody transmission in breeder Japanese quail under heat stress condition. Livest. Sci..

[22] Luo J., Ao Z., Duan Z., Ao Y., Wei S., Chen W., Chen X. (2021). Effects of N-Acetylcysteine on the reproductive performance, oxidative stress and RNA sequencing of Nubian goats. Vet. Med. Sci..

[23] Liu J., Ying Y., Wang S., Li J., Xu J., Lv P., Chen J., Zhou C., Liu Y., Wu Y. (2020). The effects and mechanisms of GM-CSF on endometrial regeneration. Cytokine.

[24] Camargo-Díaz F., García V., Ocampo-Bárcenas A., González-Marquez H., López-Bayghen E. (2017). Colony stimulating factor-1 and leukemia inhibitor factor expression from current-cycle cannula isolated endometrial cells are associated with increased endometrial receptivity and pregnancy. BMC Women’s Health.

[25] Chow R., Wessels J.M., Foster W.G. (2020). Brain-derived neurotrophic factor (BDNF) expression and function in the mammalian reproductive Tract. Hum. Reprod. Update.

[26] Antonson P., Apolinário L.M., Shamekh M.M., Humire P., Poutanen M., Ohlsson C., Nalvarte I., Gustafsson J.Å. (2020). Generation of an all-exon Esr2 deleted mouse line: Effects on fertility. Biochem. Biophys. Res. Commun..

[27] Wang Y., Wang Z., Yu W., Sheng X., Zhang H., Han Y., Yuan Z., Weng Q. (2018). Seasonal expressions of androgen receptor, estrogen receptors and cytochrome P450 aromatase in the uteri of the wild Daurian ground squirrels (*Spermophilus dauricus*). Eur. J. Histochem..

[28] Shooner C., Caron P.L., Fréchette-Frigon G., Leblanc V., Déry M.C., Asselin E. (2005). TGF-beta expression during rat pregnancy and activity on decidual cell survival. Reprod. Biol. Endocrinol..

[29] Giannubilo S.R., Landi B., Pozzi V., Sartini D., Cecati M., Stortoni P., Corradetti A., Saccucci F., Tranquilli A.L., Emanuelli M. (2012). The involvement of inflammatory cytokines in the pathogenesis of recurrent miscarriage. Cytokine.

[30] Bottazzi B., Bastone A., Doni A., Garlanda C., Valentino S., Deban L., Maina V., Cotena A., Moalli F., Vago L. (2006). The long pentraxin PTX3 as a link among innate immunity, inflammation, and female fertility. J. Leukoc. Biol..

[31] Song G., Bazer F.W., Spencer T.E. (2007). Differential expression of cathepsins and cystatin C in ovine uteroplacental tissues. Placenta.

[32] Zhang L., Li W., Song W., Ran Y., Yuan Y., Jia L., Liu L., Li Y., Cui S., Zhang Z. (2018). Detection of WNT2B, WIF1 and β-catenin expression in preeclampsia by placenta tissue microarray. Clin. Chim. Acta.

[33] Cai H.F., Chen Z., Luo W.X. (2014). Associations between polymorphisms of the GFI1B gene and growth traits of indigenous Chinese goats. Genet. Mol. Res..

[34] Tian X.Z., Li J.X., Luo Q.Y., Wang X., Xiao M.M., Zhou D., Lu Q., Chen X. (2021). Effect of supplementation with Selenium-Yeast on muscle antioxidant activity, meat quality, fatty acids and amino acids in goats. Front. Vet. Sci..

[35] Mortazavi A., Williams B.A., McCue K., Schaeffer L., Wold B. (2008). Mapping and quantifying mammalian transcriptomes by RNA-Seq. Nat. Methods.

[36] Bray N.L., Pimentel H., Melsted P., Pachter L. (2016). Erratum: Near-optimal probabilistic RNA-seq quantification. Nat. Biotechnol..

[37] Anders S., Huber W. (2010). Differential expression analysis for sequence count data. Genome Biol..

[38] Livak K.J., Schmittgen T.D. (2001). Analysis of relative gene expression data using real-time quantitative PCR and the 2(-Delta Delta C(T)) Method. Methods.

[39] Robertson S.A., Care A.S., Moldenhauer L.M. (2018). Regulatory T cells in embryo implantation and the immune response to pregnancy. J. Clin. Investig..

[40] Ashary N., Tiwari A., Modi D. (2018). Embryo implantation: War in times of love. Endocrinology.

[41] Rota C., Bergamini S., Daneri F., Tomasi A., Virgili F., Iannone A. (2002). N-Acetylcysteine negatively modulates nitric oxide production in endotoxin-treated rats through inhibition of NF-kappaB activation. Antioxid. Redox Signal..

[42] Schust D.J., Bonney E.A., Sugimoto J., Ezashi T., Roberts R.M., Choi S., Zhou J. (2021). The immunology of syncytialized trophoblast. Int. J. Mol. Sci..

[43] Mei C., Yang W., Wei X., Wu K., Huang D. (2019). The unique microbiome and innate immunity during pregnancy. Front. Immunol..

[44] Mou D., Ding D., Li S., Yan H., Qin B., Li Z., Zhao L., Che L., Fang Z., Xu S. (2020). Effect of maternal organic selenium supplementation during pregnancy on sow reproductive performance and long-term effect on their progeny. J. Anim. Sci..

[45] Mou D., Ding D., Yan H., Qin B., Dong Y., Li Z., Che L., Fang Z., Xu S., Lin Y. (2020). Maternal supplementation of organic selenium during gestation improves sows and offspring antioxidant capacity and inflammatory status and promotes embryo survival. Food Funct..

[46] Habbeddine M., Verbeke P., Karaz S., Bobé P., Kanellopoulos-Langevin C. (2014). Leukocyte population dynamics and detection of IL-9 as a major cytokine at the mouse fetal-maternal interface. PLoS ONE.

[47] Yockey L.J., Iwasaki A. (2018). Interferons and proinflammatory cytokines in pregnancy and fetal development. Immunity.

[48] Karizbodagh M.P., Rashidi B., Sahebkar A., Masoudifar A., Mirzaei H. (2017). Implantation window and angiogenesis. J. Cell Biochem..

[49] Samborski A., Graf A., Krebs S., Kessler B., Reichenbach M., Reichenbach H.D., Ulbrich S.E., Bauersachs S. (2013). Transcriptome changes in the porcine endometrium during the preattachment phase. Biol. Reprod..

[50] Li A., Li S., Zhang C., Fang Z., Sun Y., Peng Y., Wang X., Zhang M. (2021). FPR2 serves a role in recurrent spontaneous abortion by regulating trophoblast function via the PI3K/AKT signaling pathway. Mol. Med. Rep..

[51] Liu L., Wang Y., Yu Q. (2014). The PI3K/Akt signaling pathway exerts effects on the implantation of mouse embryos by regulating the expression of RhoA. Int. J. Mol. Med..

[52] Zhang X., Fu L.J., Liu X.Q., Hu Z.Y., Jiang Y., Gao R.F., Feng Q., Lan X., Geng Y.Q., Chen X.M. (2016). Nm23 regulates decidualization through the PI3K-Akt-mTOR signaling pathways in mice and humans. Hum. Reprod..

[53] Yuan L., Feng F., Mao Z., Huang J.Z., Liu Y., Li Y.L., Jiang R.X. (2021). Regulation mechanism of miR-494-3p on endometrial receptivity in mice via PI3K/AKT/mTOR pathway. Gen. Physiol. Biophys..

[54] Ji J., Chen L., Zhuang Y., Han Y., Tang W., Xia F. (2020). Fibronectin 1 inhibits the apoptosis of human trophoblasts by activating the PI3K/Akt signaling pathway. Int. J. Mol. Med..

[55] Chitu V., Stanley E.R. (2017). Regulation of embryonic and postnatal development by the CSF-1 receptor. Curr. Top. Dev. Biol..

[56] Armstrong D.L., McGowen M.R., Weckle A., Pantham P., Caravas J., Agnew D., Benirschke K., Savage-Rumbaugh S., Nevo E., Kim C.J. (2017). The core transcriptome of mammalian placentas and the divergence of expression with placental shape. Placenta.

[57] Jang H., Choi Y., Yoo I., Han J., Kim M., Ka H. (2017). Characterization of interferon α and β receptor IFNAR1 and IFNAR2 expression and regulation in the uterine endometrium during the estrous cycle and pregnancy in pigs. Theriogenology.

[58] Neirijnck Y., Papaioannou M.D., Nef S. (2019). The Insulin/IGF system in mammalian sexual development and reproduction. Int. J. Mol. Sci..

[59] Burkin H.R., Rice M., Sarathy A., Thompson S., Singer C.A., Buxton I.L. (2013). Integrin upregulation and localization to focal adhesion sites in pregnant human myometrium. Reprod. Sci..

[60] Park H.J., Park J.E., Lee H., Kim S.J., Yun J.I., Kim M., Park K.H., Lee S.T. (2017). Integrins functioning in uterine endometrial stromal and epithelial cells in estrus. Reproduction.

[61] Saeed A.M., Saenz D.J.M.L., Marco J.F., Vicente J.S. (2015). Oviductal and endometrial mRNA expression of implantation candidate biomarkers during early pregnancy in rabbit. Zygote.

[62] Long X., Zhang M., Chen X., He J., Ding Y., Zhang C., Liu X., Wang Y. (2015). Expression of KRAS in the endometrium of early pregnant mice and its effect during embryo implantation. Reprod. BioMed. Online.

[63] Cao D., Liang J., Feng F., Shi S., Tan Q., Wang Z. (2020). MiR-183 impeded embryo implantation by regulating Hbegf and Lamc1 in mouse uterus. Theriogenology.

[64] Hayashi K.G., Hosoe M., Fujii S., Kanahara H., Sakumoto R. (2019). Temporal expression and localization of vascular endothelial growth factor family members in the bovine uterus during peri-implantation period. Theriogenology.

[65] Zhao X., Du F., Liu X., Ruan Q., Wu Z., Lei C., Deng Y., Luo C., Jiang J., Shi D. (2019). Brain-derived neurotrophic factor (BDNF) is expressed in buffalo *(Bubalus bubalis*) ovarian follicles and promotes oocyte maturation and early embryonic development. Theriogenology.

[66] Arceci R.J., Shanahan F., Stanley E.R., Pollard J.W. (1989). Temporal expression and location of colony-stimulating factor 1 (CSF-1) and its receptor in the female reproductive tract are consistent with CSF-1-regulated placental development. Proc. Natl. Acad. Sci. USA.

[67] Ding J., Yang C., Zhang Y., Wang J., Zhang S., Guo D., Yin T., Yang J. (2021). M2 macrophage-derived G-CSF promotes trophoblasts EMT, invasion and migration via activating PI3K/Akt/Erk1/2 pathway to mediate normal pregnancy. J. Cell Mol. Med..

[68] Chen Q., Zhang Y., Lu J., Wang Q., Wang S., Cao Y., Wang H., Duan E. (2009). Embryo–uterine cross-talk during implantation: The role of Wnt signaling. Mol. Hum. Reprod..

[69] Poggi L., Casarosa S., Carl M. (2018). An Eye on the Wnt Inhibitory Factor Wif1. Front. Cell Dev. Biol..

[70] Tan J., Paria B.C., Dey S.K., Das S.K. (1999). Differential uterine expression of estrogen and progesterone receptors correlates with uterine preparation for implantation and decidualization in the mouse1. Endocrinology.

[71] Hummitzsch K., Hatzirodos N., Irving-Rodgers H.F., Hartanti M.D., Perry V., Anderson R.A., Rodgers R.J. (2019). Morphometric analyses and gene expression related to germ cells, gonadal ridge epithelial-like cells and granulosa cells during development of the bovine fetal ovary. PLoS ONE.

[72] Li S., Winuthayanon W. (2017). Oviduct: Roles in fertilization and early embryo development. J. Endocrinol..

